# Existing barriers to utilization of health services for maternal and newborn care in rural Western Kenya

**DOI:** 10.1186/s12913-021-06847-w

**Published:** 2021-08-11

**Authors:** George Ayodo, George O. Onyango, Salome Wawire, Nadia Diamond-Smith

**Affiliations:** 1 Jaramogi Oginga Odinga University of Science and Technology, Bondo, Kenya; 2 Independent Research Consultant, Nairobi, Kenya; 3 University of California San Francisco, San Francisco, USA

**Keywords:** Unintended pregnancy, Utilization of newborn care services, Community, health care workers.

## Abstract

**Background:**

Understanding the existing barriers to utilization of maternal and newborn health care services can inform improvement of care services in the rural settings in sub-Saharan Africa. However, how unintended pregnancy relates to the uptake of antenatal care (ANC) services and also how gaps in the role of the community health workers and health facilities affect maternal and newborn care and referral services are poorly understood.

**Methods:**

This was a formative ethnographic study design to determine barriers to the utilization of health care services for maternal and newborns in rural Western Kenya. We interviewed 45 respondents through in-depth interviews in rural Bondo Sub- County, Western Kenya: Mothers and Fathers with children under 5 years), 2 Focus Group Discussions (FGDs) with Traditional Birth Attendants (TBA), and 2 FGDs with Skilled Birth Attendants (SBAs). The data were analyzed using Atlas-ti.

**Results:**

We found that unintended pregnancy results into poor uptake of antenatal care (ANC) services due to limited knowledge and poor support system. The respondents appreciated the role of community health workers but poor government infrastructure exists. Also, perceived harshness of the health care providers, poor management of high-risk pregnancies, and unavailability of supplies and equipment at the health facilities are of concern.

**Conclusions:**

The findings of this study highlight barriers to the utilization of maternal and newborn services that if addressed can improve the quality of care within and outside health facilities.

## Background

The greatest risk to maternal and new-borns in low and middle-income settings is at the onset of labour and the first week after delivery [1–3]. As a result of limited care for the mothers and new-borns, the annual deaths for maternal and neonatal (still birth and early neonatal) stands at 280,000 and 4 million respectively in low and middle income settings [4]. However, a reduction of under-five mortality has been observed but the concern is with the slow decline of new-born mortality rate despite the availability of basic interventions [5–7]. Globally, reducing preventable morbidity and mortality of mothers and new-born is an important target in the Sustainable Development Goals but socio-cultural and economic factors remain obstacles to optimum utilization of maternal and new-born health services [8, 9]. Other contributors to morbidity and mortality are the slow adoption of deliveries at the health facilities [4, 10] and strengthening health systems [11].

Antenatal care (ANC) prevents most of the risk factors to both the mother and new-born, and related services are available virtually in all countries [12–14]. In particular, a study has shown that utilization of at least one ANC visit by skill health care provider reduces the risk of new-born mortality by 39% in sub-Saharan Africa countries [15, 16]. Another study in Ethiopia has demonstrated that at least one ANC averts about three fourth of maternal near-miss events [17].However, gaps in the use of ANC utilization persist, particularly in adherence to recommendations [18] and delay in the initiation of ANC [19]. The support of the household members to the expectant mother is very critical for complete attendance of ANC services but literature remain very limited and therefore the role of husbands, fathers and mother or grandmothers remain poorly understood.

Additionally, the community health workers (CHWs) are the first point of contact for interventions and provide essential link to health services and indeed the CHWs have been involved in promoting behaviour change through health education, early case identification and timely referral to the health facilities [20]. Due to their role in the community, care of mothers and new-born has improved the knowledge on risk and management [21] . Nevertheless, with the low birth rate at the health facilities, there is a dire need to improve their roles especially in motivation for women to change behaviour surrounding birth, delivery and new-born care [22, 23]. More importantly, there is a need to improve their services by inclusion into the government infrastructure [24, 25].

Skilled health care delivery at the health facility is an effective strategy to reduce maternal and newborn deaths. For the newborn care, there is a need for warmth, feeding support, safe oxygen use, prevention of infections etc. [26] and for the maternal, there is a need for respectful maternity care [27, 28] and proper referral guidelines especially for high-risk pregnancy to a tertiary centre of prenatal care [29]. However, gaps still exists at the rural health facilities especially on the maternal care and cost-effective low-tech equipment for newborn care [26, 30, 31] and implementation of the referrals. The aim of this study was therefore to understand barriers to utilization of maternal and newborn health care services in a rural setting.

## Method

This qualitative study was carried out between August and November 2017 in a rural community living along the shores of Lake Victoria in Siaya County, Western Kenya. The study population was selected from households surrounding three health facilities namely; Uyawi, Usigu and Got Agulu health centres in Bondo Sub County. The choice of the area was informed by previous studies that had indicated poor use of antenatal services, frequent preterm and infant mortality incidences in the community [32, 33]. After getting informed consent and assent, the participants were recruited from the community through the leaders, community health volunteers and the health workers attached to the health facilities. The interviews were scheduled by the research assistants and conducted in a private room at the health facilities at appointed times that were convenient to the participants.

Data collection was preceded with a piloting phase under which the reliability and validity of the instruments of data collection were determined. In-depth interviews were conducted with 45 participants evenly drawn from adolescent mothers, mothers and fathers, all who had children under 5 years and had some experience with preterm birth. Additionally, 6 Focus Group Discussions (FGDs) were held with a total of 60 participants in the categories of SBA, TBAs and Community Leaders, in groups of about 10. The IDIs and FGDs were conducted by research assistants under the guidance of the investigators. The interviews were done in English or Kiswahili or Dholuo depending on which language was preferred by the respondents. The data was collected through note taking, audio recording and photography. The use of IDIs and FGDs provided a process in which the data collected was verified and triangulated through varied sources. The listed names are not the real names of the study participants. The data collected was transcribed and translated from Dholuo to English before being read and cleaned.

The data was analyzed using Atlas-ti Version 7.5.7 Software [34]. We used a grounded theory approach to analyze the data, where codes were identified a priori and after that, themes were formed from these codes. Throughout the coding process, additional code or themes that emerged spontaneously during the course of the interviews were also included into our codebook. A codebook was developed through an iterative process with four team members (all authors). Codebooks had a similar foundational structure for all types of respondents (mothers, fathers, community health workers), however, different codes were added where needed to represent different themes that arose among different participant-types. Additional codes were added to the focus group discussions to highlight relationships/interactions between participants. Once the codebook was finalized, the multi-country team coded the transcripts, with over 10% double-coded. Discrepancies between the coders were discussed and resolved and we worked together in an iterative manner until inter-coder agreement exceeded 80% (indicating high levels of coder agreement) [35]. We used inductive and deductive approaches to identify the themes from the codes. Themes were developed and content analysed both within respondent type (for example, within all women) and across respondent type (looking at husband, women and health care workers).

This study received human subjects approval from the Jaramogi Oginga Odinga Teaching and referral Hospital, Kisumu Kenya and the University of California, San Francisco, USA. This study is nested in a larger study that focused on understanding and informing an intervention to improve knowledge and perception about the menstruation, preterm births and related care seeking practices in a rural settings.

## Results

### Demographic characteristics of participants

The Table 1 summarizes the socio-demographic characteristics of the study participants. The average age of women was 24.8 years, adolescent mothers was 17.9 years while for men was 35.2 years. Men had higher educational levels over 50% having attained secondary education and above while about only 40% of women had secondary and tertiary education. In addition, men had higher average number of children at 3.3 than adult women and adolescent mothers who had 2.1 and 1.3 respectively.

**Table 1:** Participants’ Demographic Summary

Parameter	Women (***n*** = 15)	Teenage Mothers (*n* = 15)	Men (*n* = 15)
**Age** (mean, range)	24.8, 20–31	17.9, 16–19	35.2, 24–55
**Education**			
Primary	60% (*n* = 9)	60% (*n* = 9)	46.7%(*n* = 7)
Secondary	26.7% (*n* = 4)	40% (*n* = 6)	46.7% (*n* = 7)
Tertiary	13.3% (*n* = 2)	0	6.7% (n = 1)
**Number of Children** (mean, range)	2.1, 1–3	1.3, 1–3	3.3, 1–6

### Socio, cultural and knowledge barriers to utilization of ANC services: adolescent mothers perspectives

Utilization of ANC services by adolescent women who often have unintended pregnancies was impacted by the support of the family members and role of the mother of an expectant adolescent girl. We observed that if an unintended pregnancy was out of the wedlock, the father of the child was expected to offer support. However, poor communication was a potential cause of delay for ANC services.

I: *Why did you decide to start clinic visits at month 4?*

*R: My mother kept insisting that the man who impregnated me should offer support. I went and looked for his phone number and called him. He advised me to start my clinic visits.***(Achieng’, 16years, Single, mother of one)**

Other adolescent respondents described how poor knowledge of their pregnancy status could result into delay of ANC attendance.

*R: I also discovered when I felt the baby kicking; babies usually start kicking at 4 months.*

*I: so when you discovered, was that when you first started going to the clinic?*

*R: I started at 6 months*

*I: why is that?*

*R: [giggling] some people even start clinic visits at 8 months and they finish*

**(Adhiambo, 17years, single, mother of one)**

For the expectant adolescent girls under care of their mothers, we observed that their mothers play key role on when to start attending ANC and therefore poor knowledge of the mothers of adolescent women affects ANC attendance.

I*: what prevented you from starting clinic visits at 3 months?*

*R: I thought that it may not be pregnancy after all. I asked my mother and she advised me to start my clinic visits at 4 or 5 months*.

**(Linda, 19years, Single, Mother of one)**

### Past experiences and economic barriers to utilization of ANC services: mothers’ perspectives

Some older women start late because of their economic status. For example, those that live far from the health facility and could not afford to pay the costly fare for all the visits.

*I: how long did you take before you started clinic visits? At how many months did you start clinic visits?*

*R: 5 months*

*I: why didn’t you go to the clinic between months 2 and 5?*

*R: sometimes you come from very far, and ideally you should be going to the clinic every month. You may not have fare for and yet you have to board a motorbike or vehicle. That is why I said I could start clinic visits from that time because sometimes I was tired and I did not have fare. That is why we start clinic visits late.*

**(Dora, 28 years, married, mother of 3)**

Other delays in ANC were due to negative previous experiences in health facilities. For instance, a respondent with a past experience of a harsh health worker explained that this made her delay to go for ANC services.

*R: I went later when the pregnancy was 5 months*

*I: Can you share with me what made you take that long before going to the clinic?*

*R: I was afraid because of what I saw in a certain clinic in Kibera ….they were very harsh*

**(Mona, 25years, Married, Mother of three)**

Getting medication from a pharmacists/chemist without appropriate health information received from the ANC services results in use of both conventional and herbal drugs, as well as general lack of knowledge/empowerment about health.

*I: After you stopped using that family planning method how long did you take before you could conceive again?*

*R: I stayed for 1 year, during that time there were some drugs that I was using, I was told to use them so that they clean my uterus*

*I: Where did you get these drugs from?*

*R: I went to a chemist and explained my problem where I was given some capsules*

*I: For how long did you take the capsules before you got pregnant?*

*R: I used to take both the capsules and some traditional herbs*

**(Auma, 21 years, married, mother of two)**

### Barriers to utilization of services at the health facility: poor quality services

The services expected to be available at the health facilities include health care providers resuscitation skills to save the lives of preterm babies and availability of other essential equipment and services. One SBA described limited resuscitation skills and resources for supporting preterm births, which, as described below, impacted mothers and father’s decisions to seek care:

Many of the community health facilities were ill-equipped and did not have adequate preparedness to handle emergencies. It emerged that this influenced some of the community members’ decisions on health seeking behaviours during childbirth.

I: *But did this impact you and your wife’s decision about where to deliver your* *baby?*

*R: It is true because we used to visit Got Agulu Health Centre as a district health*

*Centre, but again we were worried because the facilities which were there could*

*not support her in case of emergency. So we decided to look for other options.*

**(Okumu, 35years, Married, Father of five)**

### Barriers to referrals: women’s and community health workers perspectives

Early identification of high risk pregnancies was important for timely referral and safe delivery. It however emerged that some mothers lacked understanding of why they had to be referred to a bigger health facility. For instance, one of the mothers interviewed had no information on their referrals.

*I: You have also said that the baby was taken to Bondo to be kept inside cotton*

*wool, I think the cotton wool could be bought here and that done from here*

*R: Am not sure about that, I was just hearing that the baby is being taken to Bondo*

*to be kept inside cotton wool but personally I have never seen it but it’s a common term.*

**(Auma, 21years, married, mother of two)**

It emerged that due to lack of adequate resources some referrals were delayed, thus endangering mothers with birth complications. According to one SBA, delay of referral was common in the among health facilities due to lack of ambulances and other emergency resources.

*R4: another challenge is referral. Sometimes you find that the mother delivered*

*and the time taken to refer the mother to where the child could get the best*

*healthcare takes sometimes. There is that delay because of poor referral*

*M: how can that be improved?*

*R4: buying many ambulances [laughter from the group]*

**(Nancy, 29 years, SBA, MoH)**

Another respondent was aware of her high risk pregnancy and that she was not being referred to the relevant health care services, making her decide to visit the facility several times in search of the health care.

*I: You said you had some miscarriages. When you were pregnant after that, did you worry that would have a preterm baby?*

*R: yes, I was worried.*

*I: why were you worried?*

*R: I knew I would lose the pregnancy like I lost the others. That is why I was worried.*

*I: what did you do because of the worry?*

*R: I went to hospital and told them about what had happened before. I maintained my clinic visits.*

**(Amondi, 27 years married, mother of two)**

### Opportunities and barriers for improved care: community health worker’s perspectives

One SBA respondent pointed out the important role played by the CHWs in mobilizing and sensitization of the community on health issues through training.

*I: who do you think can teach these women about what to do when they are on their period?*

*R: just those doctors or experts on women’s issues.*

*I: where would the doctor or these skilled people find these women in the community?*

*R: a CHV can be sent …the CHVs can teach them and when these skilled people (doctors and nurses) show up they can mobilize the women and gather them.*

**(Awuor, 35 years, SBA, MoH)**

Another respondent, also an SBA, suggested that care for the preterm start by empowering women with knowledge through peer training and also involving the family members. This points out some of the roles that are be played by the CHWs.

*It will not be upon the preterm only, we now have to extend to the mother and*

*psychologically prepare her and go through with the mother throughout the*

*period of taking care of the preterm baby until he is able to live in a normal*

*atmosphere. So you have to bring in the other family members so that they are*

*able to know how to take care of the preterm child.*

**(Anita, 36years, SBA, MoH)**

The community health worker links the households and health facilities, however, we found gaps on their motivation with respect to their irregular payment. According to a respondent who is SBA, CHWs should be empowered and motivated.

*M: how can we improve the issue of home delivery for preterm births?*

*R12: advocacy*

*R4: the CHWs should be empowered and motivated so that they can go round. They are not motivated; they have not been paid for the last 5 months*

**(Mary, 40 years, SBA, MoH)**

## Discussions

We observed that unintended pregnancy results into poor uptake of ANC services due to limited knowledge and poor support from the family. Indeed, our study shows that husbands and mothers of expectant adolescent women make misinformed decisions that results into the delay to seek ANC services. Our finding is in agreement with previous studies have associated unintended pregnancy and adolescent pregnancy with low uptake of ANC services [14, 36]. Other studies have shown a key role of husbands when they accompany their wives to seek maternal and new-born care services [37] and also when seeking antenatal and intrapartum care services [38]. However, the challenges that women with unintended pregnancies or without a partner go through from the onset of pregnancy to the delivery and early stages of new-born care have not been documented and our findings demonstrate some gaps.

Our respondents appreciated the role of the community health workers especially on bridging the gaps between health facilities and the households, but challenges remain. Since the government outlawed the TBAs, the CHWs have taken over the role of being health advisors and the immediate interveners at the household levels. Some of the roles that should be played by the community health workers are still being played TBAs, who legally are not supposed to be providing care. This is because the services offered by TBAs are available in the rural settings, while trained providers are not. Previous studies have shown that gaps exists because there are no clear governance structures to enable community health care workers play a key role on new-born care services, in particular, health promotion services [24, 25].

Our findings also show poor preparedness of the health facilities and that women fear utilizing the health care services due to attitude of the health workers. This finding is consistent with findings of several studies in sub-Saharan Africa, in particular western Kenya, at our study area or region [39, 40] [27, 28]. Lack of essential equipment such as incubators, generator etc. was also observed but existing literature show that this cuts across several countries including some middle income countries such as South Africa [26]. Interestingly, a study in Burundi provided a way to improve on the birth outcomes of preterm babies through staff training, standardized protocols, simple but essential equipment, provision of complementary a Neonatal Intensive Care Unit and Kangaroo Mother Care units, and integration of the neonatal services with emergency obstetric care in absence of high tech equipment and specialist neonatal physician staff [31]. However, the approach has not been explored in many countries. Also, our studies shows that women with high risk pregnancy are not adequately informed on the possible birth outcomes when they are referred to tertiary hospitals and this points to a gap in referral guidelines [41, 42] and limited effort to avail low tech equipment at the primary care facilities [29].

Our study adds to the literature, highlighting a need to improve utilization of ANC services by targeting expectant adolescent girls. We further point out that community health workers are key players in the primary health care and there is a need to improve on their administrative structure. In addition, we advise on the preparedness of health facilities by use of low-tech equipment not only to reduce the volume of referrals but also to provide services closer to women with high risk pregnancies.

There is a growing body of evidence on mistreatment, disrespect and abuse (also called respectful maternity care or person-centred care) during childbirth globally, including in Africa, and the role that this has in making women not want to deliver in health facilities. Less research has focused on respectful care during ANC visits and the impact that this might have on women wanting to attend ANC, and potentially deliver in a facility eventually. We find that fear of mistreatment is a barrier to ANC use, especially by adolescents or those with unintended pregnancies. Expanding the broader literature to include mistreatment during ANC, and also expanding interventions addressing respectful care to target ANC providers as well is critical.

One limitation our study is that data were collected from a single ethnic group in Kenya and therefore views may not be representing all women and men in Kenya. Data was collected from women and men who had experienced a preterm birth, and thus over-represents the experiences and views of this population. However, this population is understudied and at high risk of not receiving adequate prenatal or delivery care, and thus their barriers are important to understand. Additionally, all respondents had to have given birth in the previous 5 years, and thus some births might have happened a few years ago and respondents may have suffered from recall bias. The respondents in the FGDs had different educational and socio-economic levels, especially traditional birth attendants, which might have made interactions uncomfortable. Also due to past experience, other skilled birth attendants some were more informed than others, and this could make other respondents less active during the focus group discussions. We further note that since this study was nested under a bigger study, we could not determine how other factors such as distance to the health facility, marital status contributed to the utilization of health services.

## Conclusions

We have identified barriers to utilization of maternal and newborn health care services. To mitigate new born mortality rates, there is a need to target women with unintended pregnancies to improve their uptake of ANC services. We further recommend exploring health promotion through CHWs by improving their administrative structures to strengthen the link between households and the health facilities. Finally, we recommend the adoption of low-tech equipment at primary care health facilities and improvement of referral systems with clear guidelines. Focusing on these recommendations will improve framework and strategies for implementation of new WHO standard for quality of care for newborns within and outside health facilities.

## Data Availability

Data is not publicly available due to the qualitative, personal nature of the data, however if someone is interested in accessing it, they can contact Dr. George Ayodo (gayodo@gmail.com) who can share anonymized transcripts.
